# Pharmacogenomic Testing and Patient Perception Inform Pain Pharmacotherapy

**DOI:** 10.3390/jpm11111112

**Published:** 2021-10-29

**Authors:** Feng-Hua Loh, Brigitte Azzi, Alexander Weingarten, Zvi G. Loewy

**Affiliations:** 1 Touro College of Pharmacy, New York, NY 10027, USA; feng-hua.loh@touro.edu (F.-H.L.); bazzi@student.touro.edu (B.A.); 2 Comprehensive Pain Associates, Syosset, NY 11791, USA; weingartencpm@awnymedical.com; 3 New York Medical College, Valhalla, NY 10595, USA

**Keywords:** pain, pharmacogenomics, polymorphism, pharmacotherapy

## Abstract

(1) Background: Chronic pain is one of the most common reasons for individuals to seek medications. Historically, opioids have been the mainstay of chronic pain management. However, in some patient populations, opioids fail to demonstrate therapeutic efficacy, whereas in other populations, opioids may cause toxic effects, even at lower doses. Response to pain medication is affected by many factors, including an individual’s genetic variations. Pharmacogenomic testing has been designed to help achieve optimal treatment outcomes. This study aimed at assessing the impact of CYP2D6 pharmacogenomic testing on physicians’ choice in prescribing chronic pain medications and patient pain control. (2) Methods: This retrospective study reviewed 107 patient charts from a single site pain management center. All 107 patients received pharmacogenomic testing. The outcomes of interest were confirmation that the optimal pain medication is being administered or a change in the chronic pain medication is warranted as a result of the pharmacogenomic testing. The main independent variable was the pharmacogenomic test result. Other independent variables included patient gender, race, and comorbidities. The retrospective study was reviewed and approved by the Touro College and University System IRB, HSIRB1653E. (3) Results: Patients self-reported pain intensity on a scale of 1–10 before and after pharmacogenomic testing. Then, 100% of patients in the retrospective study were tested for their pain pharmacogenomic profile. Of the 107 patients participating in the study, more than 50% had their medications altered as a result of the pharmacogenomic testing. The percentage of patients with intense pain were decreased post-pharmacogenomic testing (5.6%) as compared to pre-pharmacogenomic testing (10.5%). Patients with intense, moderate, and mild pain categories were more likely to receive changes in pain medications. In contrast, patients with severe pain were less likely to receive a change in pain medication. Hispanic ethnicity was associated with a statistically significantly decrease in a pain scale category. Illegal drug abuse was associated with a decrease in pain scale category. Change in medication dose was associated with a decrease in pain scale category. (4) Conclusion: In this retrospective study, implementation of pharmacogenomic testing demonstrated significant benefits to patients with intense pain undergoing treatment.

## 1. Introduction

Opioid analgesics are commonly prescribed to manage chronic pain. In 2019, the overall opioid prescription rate in the United States was 46.7 prescriptions per 100 people (Centers for Disease Control and Prevention). Although the prescription rate is strikingly high, the response to pain using opioid analgesics is highly variable [1]. The response variability is believed to be correlated with genetic polymorphisms [2].

Adverse side effects resulting from opioid treatment are very prevalent [3]. The side effects include nausea, vomiting, respiratory depression, and addiction. Opioid addiction variability is differentiated based upon genetics [4].

Pain therapy pharmacogenomics is segmented broadly into two components. In the first component, genetic information used to examine the impact of genetic variability on factors modulating pain, and its intensity is referred to as functional pain genomics [5]. Functional pain genomics focuses on how a group of genes work in concert to regulate response to pain [6]. The second component of pain pharmacogenomics is focused on characterization of the genetic variations that contribute to an individual’s response to drugs used in pain management practice. It is pharmacogenomics of pain management that represents the most common segment of pain genomics [6].

The molecular basis for the differential response to therapeutic intervention is correlated with different forms of genetic variants. There are several types of genetic variants such as single nucleotide polymorphisms (SNPs) that are single base variations in a DNA sequence and short repeat units, short tandem repeats (STRs) that include the micro- and mini-satellites, insertions, deletions, and chromosomal aberrations [7].

Thus, the challenge for effective pharmacotherapy in pain management is clearly to provide the medication that is most appropriate for the individual patient. Using a personalized patient care approach leverages the patient’s phenotype and genotype data to define the optimal treatment approach. *CYP2D6* is a highly polymorphic gene. *CYP2D6* alleles have been characterized in several geographical and ethnic groups [8,9]. The *CYP2D6* gene product is responsible for the metabolism of approximately 25% of all currently marketed pharmaceuticals. Studies have suggested that *CYP2D6* is the primary gene implicated in the metabolism of analgesics [10]. The Clinical Pharmacogenetics Implementation Consortium (CPIC) guidelines for *CYP2D6* genotype and codeine therapy was recently updated [11]. For *CYP2D6* poor metabolizers, codeine and tramadol should be avoided, and alternative analgesics should be used instead. In this study, a retrospective chart review of chronic pain patients was used to ascertain the impact of *CYP2D6* pharmacogenomic testing on pain pharmacotherapy in concert with patient perception. Use of a personalized medicine approach could be a key enabler in curtailing the opioid crisis.

## 2. Materials and Methods

### 2.1. Study Design and Data Source

This study was a retrospective chart review using charts collected from one pain management clinic in the State of New York. The dates of the charts ranged from 15 May 2015 to 21 June 2016. Study subjects’ age, gender, race/ethnicity, type of health insurance, type of pain medication, including name, dose, dosing frequency, and administrative route, whether used pre- and/or post-pharmacogenomics testing, number of concomitant medications used, and pharmacogenomic testing results were extracted from the charts. Patients that presented with chronic pain were initially prescribed pain medication. Subsequently, pharmacogenomic testing enabled by Next Generation Sequencing (NGS) was performed at Molecular Diagnostics Laboratories, Covington, KY and the clinician used the *CYP2D6* pharmacogenomic results to make changes to the patients’ drug(s), dose, and or delivery method. Patient input was utilized to determine optimal pharmacotherapy. The primary outcome of interest was change in pain medication, including the addition of a new pain medication or discontinuation of the previous pain medication. The secondary outcome of interest was change in dose, dosing frequency, or administrative route for those prescriptions without changes in pain medication.

### 2.2. Data Analysis

After extracting the data from the charts, descriptive analysis was performed on the data. A T-test was used to test the difference in pain medication used and pain scale pre- and post-pharmacogenomics testing. For multivariable analysis, ordinary least square (OLS) regression was performed to examine the effect of change in pain medication informed by pharmacogenomic testing results as determined by pain scale, and among those whose pain medication did not change, the effect of change in dose, dosing frequency, and administrative route informed by pharmacogenomic testing results as determined by pain scale while adjusting for other factors. The alpha level was set at 0.05 for this study. All the analyses were performed using SAS.

The study was approved by the Touro College and University System IRB HSIRB1653E.

## 3. Results

A total of 107 study subjects, whose charts were reviewed, were included in this study. Table 1 presents the demographics of the study subjects included in the study. The mean age of the study subjects was 55, with a standard error of 0.13. The gender distribution of the study subjects was even (46.7% female and 53.3% male). In terms of race/ethnicity, the study subjects were predominately White (85%), followed by Black (9.3%), Asian (2.8%), Hispanic (1.9%), and others (0.9%). In terms of the type of health insurance that the study subjects had, the study subjects (51.4%) had either private health insurance, worker’s compensation insurance (45.7%), or no health insurance (2.8%).

All study subjects were prescribed opioids, along with other pain medications (Table 2). Among the non-opioids, non-steroid anti-inflammatory drugs (NSAIDs) were the most commonly prescribed (54.2% pre-PGx testing vs. 61.7% post-PGx testing), followed by acetaminophen (46.7% pre-PGx testing vs. 43.9% post-PGx testing), benzodiazepines (14.9% pre-PGx testing vs. 15.9% post-PGx testing), muscle relaxants (14.9% pre-PGx testing vs. 20.6% post-PGx testing), anesthetics (4.7% pre-PGx testing and post-PGx testing), and GABA analogs (14.9% pre-PGx testing vs. 21.5% Post-PGx testing). During the course of investigation, the study subjects took on an average of an additional 1.35 (SD = 0.012) other concomitant medications in addition to their main pain medication (data not shown).

Many of the most commonly prescribed opioids are converted by CYP2D6 to a metabolite that exhibits more potent opioid effects as compared to the parent compound. However, 5–10% of individuals have null *CYP2D6* alleles. These individuals lack enzyme activity and correspond to the *CYP2D6* poor metabolizer (PM) phenotype [11]. Table 3 summarizes the data that correspond to the primary endpoints: addition of or discontinuation of pain medications. Table 4 addresses the secondary endpoints including changes in the dose of a drug, frequency of administration, and/or the route of administration.

Pharmacogenomic (PGx) testing resulted in significant changes in pain medication. There were 347 prescriptions for pain medications pre-PGx testing. Among the 347 prescriptions, 115 (33.1%) were discontinued, 58 (16.7%) were maintained but with a change in dose, administrative route, or frequency, and 174 (50.1%) remained the same post-PGx testing. After PGx testing, 70 new prescriptions for pain medications were added.

Table 5 presents the pain scale results pre- and post-pharmacogenomic testing. There were missing values for 11 study subjects pre- and post-PGx testing. The initial pain scale used on the charts ranged from 1 to 10. The mean pain scale was 7.25 (SD = 2.04) pre-PGx testing and 7.00 (SD = 2.07) post-PGx testing. To better understand where the change in pain assessment between pre- and post-PGx testing occurred, we further categorized the pain scale as mild pain (1–3), moderate pain (4–6), severe pain (7–9), and intense pain (10). For both pre-PGx testing and post-PGx testing measures, most of the study subjects (59.0% pre-PGx testing and 61.9% post-PGx testing) had severe pain. The proportion of study subjects with intense pain decreased from pre-PGx testing (10.5%) to post-PGx testing (5.7%), whereas the proportion of study subjects with severe pain and moderate pain increased (non-significantly) from pre-PGx testing (59.0% and 23.8%, respectively) to post-PGx testing (61.9% and 25.7%, respectively). The proportion of study subjects with mild pain remained the same pre- and post-PGx testing (6.7%).

Table 6 shows patients’ pain scale pre- and post-PGx testing, stratified by change in medication post-PGx testing. Eighty percent of the study subjects received a change in their pain medication post-PGx testing. Patients with intense pain (83.33%), moderate pain (81.48%), and mild pain (85.71%) were more likely than average to receive a change in their pain medication, while those with severe pain (78.46%) were less likely than average to receive a change in their pain medications post-PGx.

Table 7 shows the effect of change in medication post-PGx testing on patient pain scale while adjusting for other factors. Change in medication post-PGx testing was associated with a decrease (−0.02, 95% Confidence Interval (CI) −0.3015–0.2582) in pain scale category. Hispanic ethnicity (two patients) was associated with a statistically significant decrease (−0.78, 95% CI −1.6430–0.0681) in pain scale category. Illegal drug use (three patients) was also associated with a statistically significant decrease (−0.67, 90% CI −1.3774–0.0296) in pain scale category.

Table 8 shows the effect of change in dose post-PGx testing on patient pain scale among those who did not receive any change in medication post-PGx testing while adjusting for other factors. Change in dose post-PGx testing was associated with a decrease (−0.66, 90% CI −1.1380–0.1831) in pain scale category. Having worker’s compensation insurance was associated with a statistically significant decrease (−0.37, 90% CI −0.7129–0.0351) in pain scale category.

The effects of changes in dosing frequency and administrative route on patient pain scale among those who did not receive any change in medication post-PGx testing were not statistically significant and therefore not presented.

## 4. Discussion

The study of pain is challenging. Pain is complex; it is influenced by genetic traits, race, ethnicity, and gender [12,13,14,15,16]. More than 200 genes have been implicated in pain processing [13]. Several hereditary disorders are associated with pain insensitivity including Lesch–Nyhan syndrome, de Lange syndrome, and Tourette’s syndrome [13]. Genetics contributes to migraines, lower back pain, and menstrual pain [13].

Racial and ethnic disparities in pain have been extensively reported [13,14,15,16]. African Americans report sensitivity to pain greater than that of Caucasians for migraines, headaches, orofacial pain, postoperative pain, and arthritis and joint pain [16]. Twenty-eight percent of Hispanics over the age of 50 report having consistent pain [17]. The sensitivity to and perception of pain is compounded in that reports suggest lower quality of care is administered to patients who are racial and/or ethnic minorities including Hispanics and African Americans, irrespective of whether the treatment is for acute pain, chronic pain, cancer pain, or palliative pain care [18,19].

The literature suggests that men and women differ in their responses to pain, with increased pain sensitivity observed among women [12]. Several common chronic pain conditions are greater for women than men, including fibromyalgia, migraine and chronic tension-type headache, irritable bowel syndrome, temporomandibular disorders, and interstitial cystitis [20,21,22,23].

This study demonstrated the successful implementation of pharmacogenomics in guiding the pharmacotherapy of chronic pain patients. The use of pharmacogenomics was deemed successful as changes in pharmacotherapy exhibited improved patient pain control in the intense pain population. The changes made in pharmacotherapy were both in the drugs administered as well as in the dose of drugs.

This investigation delivered six key outcomes: (1) Pain medication distribution was changed by the physicians during the course of the study. As a result of pharmacogenomic testing, 70 new medications were added, 115 medications were discontinued, and 58 medications had changes made in dose administered. (2) The percentage of patients with intense pain were decreased post-pharmacogenomic testing (5.6%) as compared to pre-pharmacogenomic testing (10.5%). (3) Patients with intense, moderate, and mild pain categories were more likely to receive changes in pain medications. In contrast, patients with severe pain were less likely to receive a change in pain medication. (4) Hispanic ethnicity was associated with a statistically significantly decrease in a pain scale category. (5) Illegal drug abuse was associated with a decrease in pain scale category. (6) Change in medication dose was associated with a decrease in pain scale category.

## 5. Conclusions

When treated for pain, variation in response to medication exists across populations. Genetic factors are a factor in the response differential. Our study demonstrates that pharmacogenomic testing has the potential to improve therapeutic outcomes. Further studies investigating the contribution of phenoconversion as well as drug–drug interactions are warranted [24]. Improvement in individual’s quality of life will be a corollary to the improvement of therapeutic outcome.

## Figures and Tables

**Table 1:** Patient characteristics (N = 107).

Demographics	N (%)
Age (year) (mean, ±SD)	54.76 (0.13)
Gender	
Female	50 (46.7)
Male	57 (53.3)
Race/ethnicity	
White	91 (85)
Black	10 (9.3)
Hispanic	2 (1.9)
Asian	3 (2.8)
Others	1 (0.9)
Type of health insurance	
Private	54 (51.4)
Worker’s Comp	48 (45.7)
No insurance	3 (2.8)
Tobacco use	20 (18.7)
Personal history of substance abuse	
Alcohol	7 (6.5)
Illegal drugs	3 (2.8)
Prescription drugs	15 (14.0)
Psychological disorder	
Attention deficit disorder (ADD)	6 (5.6)
Obsessive compulsive disorder (OCD)/bipolar/schizophrenia	6 (5.6)
Depression	24 (22.4)

**Table 2:** Type of pain medications prescribed to patients pre- and post-pharmacogenomic (PGx) testing (N = 107).

Type of Pain Rx	Pre-PGx Testing (%)	Post-PGx Testing (%)
Opioids	107 (100)	107 (100)
NSAIDs	58 (54.2)	66 (61.7)
Acetaminophen	50 (46.7)	47 (43.9)
Benzodiazepines	16 (14.9)	17 (15.9)
Muscle relaxant (carisoprodol)	16 (14.9)	22 (20.6)
Anesthetic (lidocaine)	5 (4.7)	5 (4.7)
GABA analog (pregabalin, duloxetine)	16 (14.9)	23 (21.5)

**Table 3:** Change in pain medication post-CYP2D6 pharmacogenomic testing, stratified by phenotype.

Phenotype	Total Number of Rx	Added New Rx	Discontinued Old Rx	No Change
Ultrarapid metabolizer	5	3	0	2
		(60.0%)	(0.0%)	(40.0%)
Normal metabolizer	254	29	87	138
		(11.4%)	(34.2%)	(54.3%)
Intermediate metabolizer	70	25	6	39
		(35.7%)	(8.6%)	(55.7%)
Poor metabolizer	75	13	24	38
		(17.3%)	(32%)	(50.6%)

**Table 4:** Change in dose/dosing frequency in pain medication post-CYP2D6 pharmacogenomic testing, stratified by phenotype.

Phenotype	Total Number of Rx	Change in Dose ONLY	Change in Frequency ONLY	Change in Dose and Frequency	No Change
Ultrarapid metabolizer	2	0	1	0	1
		(0.0%)	(50.0%)	(0.0%)	(50%)
Normal metabolizer	138	5	20	5	108
		(3.6%)	(14.5%)	(3.6%)	(78.3%)
Intermediate metabolizer	39	2	6	1	30
		(5.1%)	(15.4%)	(2.6%)	(76.9%)
Poor metabolizer	38	3	3	4	28
		(7.9%)	(7.9%)	(10.5%)	(73.7%)

**Table 5:** Patients’ pain scale pre- and post-pharmacogenomic (PGx) testing.

Pain Scale	Pre-PGx Testing (N = 105)	Post-PGx Testing (N = 105)
Mean (±SD)	7.25 (2.04)	7.00 (2.07)
Categories		
Intense pain	11 (10.5%)	6 (5.7%)
Severe pain	62 (59.0%)	65 (61.9%)
Moderate pain	25 (23.8%)	27 (25.7%)
Mild pain	7 (6.7%)	7 (6.7%)

**Table 6:** Patients’ pain scale pre- and post-pharmacogenomic (PGx) testing, stratified by change in medication post-PGx testing.

Pain Scale	Pre-PGx Testing		Post-PGx Testing			
	N = 105 (107 − 2)		Any Change in Medication Post-PGx Testing (N = 84 (86 − 2))		No Change in Medication Post-PGx Testing (N = 21)	
Categories	N (%)	Mean (±SD)	N (%)	Mean (±SD)	N (%)	Mean (±SD)
Intense pain	11 (10.5)	10 (0)	5 (5.6)	10 (0)	1 (4.8)	10 (0)
Severe pain	62 (59.0)	8.1 (0.76)	51 (60.7)	8.0 (0.71)	14 (66.7)	8.1 (0.92)
Moderate pain	25 (23.8)	5.3 (0.79)	22 (26.2)	5.1 (0.90)	5 (23.8)	4.8 (0.84)
Mild pain	7 (6.7)	2.4 (0.53)	6 (7.1)	2.3 (0.61)	1 (4.8)	2 (0)

**Table 7:** Effect of change in medication post-pharmacogenomic (PGx) testing on patient pain scale (N = 107).

Variable	Effect
Any change in medication post-PGx testing	−0.0217 (−0.3015–0.2582)
Age	0.0025 (−0.0074–0.0125)
Gender	
Female (ref)	0.0307 (−0.2574–0.3189)
Male	
Race/ethnicity	
White (ref)	
Black	−0.0789 (−0.5379–0.3801)
Hispanic	−0.7874 (−1.6430–0.0681)
Asian	0.0820 (−1.0878–1.2518)
Type of health insurance	
Private (ref)	
Worker’s Comp	−0.0949 (−0.3635–0.1738)
No insurance	−0.8634 (−2.1391–0.4123)
Tobacco use	−0.1208 (−0.4784–0.2368)
Personal history of substance abuse	
Alcohol	0.3642 (−0.1587–0.8871)
Illegal drugs	−0.6739 (−1.3774–0.0296)
Prescription drugs	0.2231 (−0.1349–0.5811)
Psychologic disease	
ADD	0.2785 (−0.2791–0.8361)
OCD/bipolar/schizophrenia	0.3446 (−0.3604–1.0496)
Depression	0.0622 (−0.2634–0.3880)

**Table 8:** Effect of change in dose post-pharmacogenomic (PGx) testing on patient pain scale among those without change in medication post-PGx testing (N = 28).

Variable	Effect
Change in dosing post-PGx testing	−0.6605 (−1.2759–0.0452) *
Age	−0.0010 (−0.0175–0.0155)
Gender	
Male (ref)	
Female	−0.0397 (−0.5444–0.4648)
Race/ethnicity	
White (ref)	
Black	−0.2488 (−1.1060–0.6084)
Hispanic	-
Asian	-
Type of health insurance	
Private (ref)	
Worker’s Comp	−0.3740 (−0.8108–0.0628)
No insurance	−0.9050 (−2.2242–0.4142)
Tobacco use	−0.1892 (−0.8480–0.4695)
Personal history of substance abuse	
Alcohol	0.0349 (−0.8220–0.8917)
Illegal drugs	−0.5755 (−1.7689–0.6180)
Prescription drugs	0.4878 (−0.1259–1.1016)
Psychologic disease	
ADD	−0.0346 (−1.3021–1.2328)
OCD/bipolar/schizophrenia	-
Depression	−0.1050 (−0.7852–0.5752)

Note: * *p* < 0.017.
